# Advances in the diagnosis of non-occlusive mesenteric ischemia and challenges in intra-abdominal sepsis patients: a narrative review

**DOI:** 10.7717/peerj.15307

**Published:** 2023-04-26

**Authors:** Zhou Chen, Xiaosun Liu, Chunhui Shou, Weili Yang, Jiren Yu

**Affiliations:** Department of Gastrointestinal Surgery, Zhejiang University School of Medicine First Affiliated Hospital, Hangzhou, China

**Keywords:** Non-occlusive mesenteric ischemia, Sepsis, Diagnosis, Review

## Abstract

Non-occlusive mesenteric ischemia (NOMI) is a type of acute mesenteric ischemia (AMI) with a high mortality rate mainly because of a delayed or misdiagnosis. Intra-abdominal sepsis is one of the risk factors for developing NOMI, and its presence makes early diagnosis much more difficult. An increase in routine abdominal surgeries carries a corresponding risk of abdominal infection, which is a complication that should not be overlooked. It is critical that physicians are aware of the possibility for intestinal necrosis in abdominal sepsis patients due to the poor survival rate of NOMI. This review aims to summarize advances in the diagnosis of NOMI, and focuses on the diagnostic challenges of mesenteric ischemia in patients with intra-abdominal sepsis.

## Introduction

Non-occlusive mesenteric ischemia (NOMI) is defined as a subtype of mesenteric ischemia without thrombotic occlusion of blood vessels. It accounts for 20% to 30% of all causes of acute mesenteric ischemia (AMI) (Trompeter et al., 2002). It has a mortality rate of 70% to 90% (Schoots et al., 2004), mainly because of a delayed or misdiagnosis. NOMI is thought to be caused by decreased cardiac output or hypovolemia, resulting in splanchnic hypoperfusion, and is a condition that occurs primarily in critically ill patients in the intensive care unit (ICU).

According to the Third International Consensus Definitions for Sepsis and Septic Shock (Sepsis-3), abdominal sepsis is defined as an increase of the Sequential Organ Failure Assessment (SOFA) score of ≥2 points as a result of intra-abdominal infections (IAIs) (Singer et al., 2016). Abdominal sepsis is a risk factor for NOMI (Acosta et al., 2006; Kniemeyer, 1998; Stahl et al., 2020) and the mechanisms of intestinal mucosal injury caused by abdominal sepsis are diverse. Hypovolemia, high catecholamine doses, enteral nutrition (EN), and even the cellular and metabolic disorders of sepsis may result in mucosal injury (Abraham & Singer, 2007; De Backer et al., 2003; Kolkman & Mensink, 2003; Meier-Hellmann et al., 1997; Reignier et al., 2018; Singer et al., 2016). However, the predominant cause of NOMI in patients with advanced sepsis is decreased splanchnic circulation due to the constriction of mesenteric blood vessels in response to high doses of inotropes. Moreover, NOMI-induced intestinal necrosis is indicative of a secondary worsening of abdominal sepsis. A study revealed that NOMI is the second and third cause of early (≤3 d) and late (>3 d) death in septic shock patients in ICU, respectively (Daviaud et al., 2015). The early diagnosis of NOMI is essential for improving its prognosis; however, early diagnosis remains challenging, especially in intra-abdominal sepsis patients.

Here we summarized the process developed for the successful diagnosis of NOMI and focused specifically on the diagnostic challenges of NOMI in intra-abdominal sepsis patients.

## Survey methodology

We used PubMed to search for relevant studies for this literature review. The relevant keywords were: “non-occlusive mesenteric ischemia” and “diagnosis”; “mesenteric ischemia” and “diagnosis”; “non-occlusive mesenteric” and “sepsis”; “mesenteric ischemia” and “sepsis”; “NOMI” and “sepsis”. Additionally, author names and reference lists were used to further search the articles for related references.

### Clinical examination

The clinical signs of NOMI are non-specific (van den Heijkant et al., 2013). Although the most common symptom is vague abdominal pain, most abdominal sepsis patients under sedation are unable to verbalize abdominal pain or other digestive symptoms.

Clinically, ischemic lesions are first reported to appear in the intestinal mucosa and then ultimately progress to irreversible transmural necrosis (Reilly et al., 2001; Reintam Blaser, Acosta & Arabi, 2021). In the early stages, the most common symptoms are sudden abdominal pain, nausea, vomiting, and diarrhea, but without obvious abdominal signs. As the ischemic time is extended, the whole layer of the intestinal wall may become necrotic, leading to bacterial flora translocation. The development of an intra-abdominal infection may aggravate a patient’s intraabdominal pain and their temperature may rise. Peritoneal irritation signs commonly indicate the occurrence of peritonitis, intestinal necrosis, or intestinal perforation in NOMI patients. However, some studies have shown that signs of peritoneal irritation in a patient admitted to the emergency room typically led the treating physician to initially consider diagnoses other than AMI (Karkkainen, 2021; Karkkainen et al., 2015).

### Biomarkers

The most common biomarkers for detecting mesenteric ischemia are leukocytosis, metabolic acidosis, high lactate concentrations, and elevated D-dimer (Cudnik et al., 2013; Oldenburg et al., 2004). Several studies have reported the diagnostic value of D-dimer and lactate in AMI (Acosta, Nilsson & Björck, 2004; Block et al., 2008; Chiu et al., 2009; Cudnik et al., 2013; Montagnana, Danese & Lippi, 2018), but the sensitivity rate was far from satisfactory. The diagnostic properties of plasma biomarkers in the NOMI subgroup have also been considered. A study involving 25 patients with NOMI, reported by Matsumoto et al. (2019), showed that D-dimer has a sensitivity of 52% and specificity of 87.3%, and lactate has a sensitivity of 60% and specificity of 88.7% (Table 1). Hong et al. (2017) and Klingele et al. (2015) investigated the presence of NOMI after cardiac surgery (Table 1). However, high C-reactive protein (CRP) levels, leukocytosis, elevated procalcitonin, metabolic acidosis, high lactate concentrations, and elevated D-dimer are all common in sepsis patients, indicating that intra-abdominal sepsis patients may have abnormal biomarkers before the development of NOMI. Therefore, the specificity of the abovementioned classical biomarkers may be even lower in sepsis patients.

**Table 1:** Diagnostic properties of plasma biomarkers.

Biomarkers investigated	Sensitivity (%)	Specificity (%)	Area under the curve
Matsumoto et al.			
D-dimer	52.0	87.3	0.691
Lactate	60.0	88.7	0.646
I-FABP	76.0	80.3	0.805
CRP	52.0	73.2	0.647
CK	40.0	90.1	0.630
WBC	40.0	85.9	0.582
Hong et al.			
D-lactate	39.0	100	0.510
I-FABP	31.0	100	0.640
SMA	54.0	100	0.680
Klingele et al.			
PCT	71.0	94.0	0.940
Groesdonk et al.			
Endothelin-1	51.0	94.0	0.770

**Note:**

I-FABP, intestinal fatty acid-binding protein; CRP, C-reactive protein; CK, creatine kinase; WBC, white blood cell; SMA, smooth muscle actin; PCT, procalcitonin.

Intestinal fatty acid-binding protein (I-FABP) is a 15-kDa soluble protein released by mature enterocytes and associated with intestinal ischemia (Kanda et al., 1996). It was reported as a promising laboratory indicator in diagnosing intestinal ischemia (Schellekens et al., 2014; Thuijls et al., 2011). Matsumoto et al. (2019) demonstrated that I-FABP had a sensitivity of 76% and specificity of 80.3%, with an area under the curve of 80.5%, when compared to other classical biomarkers (Table 1). In septic shock patients, the I-FABP level was reported to be associated with the incidence of NOMI and was able to predict a 28-day mortality rate (Sekino et al., 2017). However, I-FABP still has a few limitations that preventing it from being a sensitive diagnostic tool in sepsis patients. Other bowel diseases such as small bowel obstruction and ulcerative colitis can elevate I-FABP (Cronk et al., 2006; Wiercinska-Drapalo et al., 2008) and the intensity and duration of shock have been associated with enterocyte injury (Piton et al., 2013). I-FABP was rapidly cleared by the kidney with a half-time of approximately 11 min (van de Poll et al., 2007; van Haren, 2013) and was reported to be significantly higher in renal failure patients than those with normal renal function (Okada et al., 2018). Therefore, it was unclear whether the elevated I-FABP levels in septic shock patients with renal insufficiency were attributed to increased generation or decreased filtration, indicating that the concentration of I-FABP may elevate in septic shock patients even if there was no NOMI. Meanwhile, a subset of NOMI patients exhibited lower I-FABP levels, potentially resulting from enterocyte depletion within the ischemic and/or necrotic bowel (Sekino et al., 2017). Researchers in the Netherlands have shown that I-FABP in urine has a better early diagnostic value than that in serum for early mesenteric ischemia (Thuijls et al., 2011). However, acute renal failure is prevalent in intra-abdominal sepsis patients and urine samples may not be available.

Hong et al. (2017) found that all patients with high amounts of smooth muscle actin (SMA) developed intestinal necrosis, but the number of patients in their study was small (*n* = 7). Another cohort study conducted by Groesdonk et al. (2015) enrolled 78 NOMI cases out of 865 patients undergoing elective cardiac surgery, suggesting that the elevated endothelin-1 serum levels had a high accuracy to predict NOMI (Table 1). The main mechanism leading to NOMI after cardiac surgery is decreased cardiac output. However, there are multiple mechanisms of NOMI caused by sepsis. Calame et al. (2021) identified that a plasma bicarbonate concentration ≤15 mmol/L and a prothrombin rate <40% were associated with a high risk of irreversible transmural necrosis (ITN) in NOMI, but the proportion of sepsis was 15% in all conditions associated with NOMI in this study. Therefore, further research is required to verify the values of those known biomarkers in the sepsis subgroup and to explore new biomarkers with better diagnostic accuracy.

### Imaging

Angiography is considered to be the historical gold standard for diagnosing cardiac issues (Trompeter et al., 2002). In 1974, Siegelman, Sprayregen & Boley (1974) depicted the angiographic criteria for the diagnosis of mesenteric vasospasm. This criteria included: (a) narrowing at the origins of the multiple branches of the superior mesenteric artery; (b) irregularities at the origins of the major branches of the superior mesenteric artery; (c) spasm of the arcades of the mesenteric artery; and (d) impaired filling of intramural vessels (Siegelman, Sprayregen & Boley, 1974). In severe NOMI cases, vasoconstriction may be present in the branches of the celiac trunk and the inferior mesenteric artery (IMA), as well as the renal arteries (Trompeter et al., 2002). However, some angiographic findings are not specific to NOMI; for instance, the narrowing of the intestinal branches may also be caused by arteriosclerosis. Angiography is also a time-consuming and invasive tool, and it may not be emergently available to critically ill patients. Therefore, it is not the tool of choice for diagnosing NOMI.

Previous studies have described biphasic contrast-enhanced multidetector computed tomography (MDCT). This method provides high-quality and multi-planar reconstructed (MPR) images that are equivalent to angiography with less invasiveness and less time for the diagnosis of NOMI (Kammerer et al., 2018; Woodhams et al., 2010). A study revealed that two patients with angiography had MPR images that were concordant with their angiograms (Woodhams et al., 2010). Bourcier et al. (2016) identified four representative CT‑scan findings of NOMI in biphasic contrast-enhanced MDCT: (a) the absence of bowel wall enhancement; (b) pneumatosis intestinalis and the absence of bowel wall enhancement; (c) bowel dilatation and the absence of bowel wall enhancement; and (d) portal venous gas. A study that reviewed preoperative abdominal enhancement CT performance in 145 patients with pathologically confirmed NOMI showed that the absence of bowel wall enhancement was the most consistent CT feature of transmural necrosis (Verdot et al., 2021). Calame et al. (2021) identified that the absence of bowel enhancement and bowel thinning was associated with ITN in the context of NOMI. Therefore, an isolated pneumatosis was not considered to be a sign of necrosis in a recent study (Calame, Delabrousse & Ronot, 2022).

The signs of abnormal intestinal wall were typically identified in the late stages of NOMI, but early-stage indicators were undefined on MDCT. A study that measured the diameter of the superior mesenteric artery during the ischemic stage and compared it to the diameter in the previous CT image (non-NOMI) of the same patient showed that the diameters of all 55 cases were smaller in the NOMI scan (Pérez-García et al., 2018). This may indicate that the morphology and diameter of the superior mesenteric artery are the critical evaluation indicators in the early stage. However, norepinephrine was recommended as a first line agent to correct hypotension during septic shock resuscitation and may cause vasoconstriction. Animal experiments have shown that cellular and molecular biology changes and metabolic acidosis caused by sepsis may actually reduce vascular contractility (Barrett et al., 2007; Kim et al., 2005; Zhang et al., 2018). Therefore, it is difficult to determine the imaging criteria for the diameter of the superior mesenteric artery that would indicate NOMI in septic shock patients.

Biphasic contrast-enhanced MDCT may still be used in a number of circumstances. These include making a differential diagnosis in occlusive ischemia and other causes of acute abdomen, evaluating the degree of bowel wall enhancement (absent when intestinal necrosis), or investigating the reperfusion event in NOMI. Reperfusion events are more common in NOMI than in the occlusive type of AMI and may determine the course of follow-up treatment (Bagnacci et al., 2021; Mazzei et al., 2016; Mazzei & Volterrani, 2015). Effective reperfusion may not require surgical intervention, but invalid or absent reperfusion may indicate the need for early surgical intervention. Angiography may represent an excellent therapeutic option and its combined use with intra-arterial vasodilator infusion at the early stages of disease has improved the mortality rate from 70–90% to 10–50% (Boley et al., 1977; Flobert et al., 2000). Notably, septic shock patients frequently have acute kidney injury and an iodinated contrast agent may cause the deterioration of the patient’s kidney function. Compared with conventional single-energy CT, dual-energy computed tomography (DECT) has been reported to gain a higher quality of superior mesenteric artery in 3D and MPR images while using a lower concentration of contrast agent (Patel et al., 2017). More studies are needed in the future to prove its value in the diagnosis of mesenteric ischemia.

### Endoscopy

Among the patients with suspected NOMI, some studies have shown that endoscopy may help with the diagnosis of NOMI when there were no positive indicators from other sources (Bourcier et al., 2016; Paul et al., 2020). Paul et al. (2020) used three grades to identify NOMI in suspected patients: grade 1, mucosal edema and erythema; grade 2, non-necrotic ulcerations on an edematous mucosa; and grade 3, necrosis with grey-black mucosal discoloration. The advantages of endoscopy are apparent. It allows the direct visualization of the intestinal mucosa and other acute abdominal diseases may be ruled out. However, its disadvantages are numerous. The small bowel, which is a large part of the intestines, is inaccessible; it lacks the sensitivity and specificity needed to detect more subtle ischemic changes; the observed ischemia changes do not indicate whether the cause of ischemia is due to occlusive or non-occlusive; the observed mucosal necrosis does not always correspond to transmural necrosis. Moreover, it was also reported that colonoscopy was one of the risk factors for colon ischemia (Sadalla et al., 2021).

In patients with intra-abdominal sepsis, some intestinal wall abnormalities, such as edematous mucosa, may exist before progressing to NOMI. These issues may interfere in obtaining the proper diagnosis. The stretch pressure of the scope against the bowel wall, over-insufflation, and barotrauma can predispose an area of weakened colonic wall to perforation due to its thinner wall and higher wall tension (Owen, 2006). The risk of perforation when colonic wall necrosis is present in intra-abdominal sepsis patients should be further studied.

### Explorative laparoscopy

Technological advances in laparoscopy are a convenient and accurate diagnostic modality for critically ill patients who need a timely abdominal evaluation. In NOMI patients with present or suspected necrosis, Cocorullo et al. (2017) found that explorative bedside laparoscopy was a feasible and safe surgical approach for necrotic intestinal detection and resection. This procedure utilizes a laparoscopic column and a centralized CO_2_ distribution system available in the ICU for the performance of a bedside laparoscopy, and could reduce the mortality rate by avoiding approximately 45% of unnecessary laparotomies (Cocorullo et al., 2017). Moreover, a recent study reported that laparoscopy has lower postoperative complication rates and wound infection incidences than laparotomy in detecting patients with a high suspicion of NOMI (Bergamini et al., 2022). Diagnostic laparoscopy is an invasive tool that is considered to be the most appropriate and most beneficial in cases of advanced ischemia or strongly suspected intestinal necrosis. Improvements in patient mortality can be observed when laparoscopy is the last diagnostic procedure and the first therapeutic modality used, and it reduces the possibility of bowel resection, avoids unnecessary laparotomy, and allows for a more tailored open surgery when NOMI is confirmed.

It is unclear whether a laparoscopic approach worsens the septic state or whether minimally invasive surgery is beneficial in critically ill patients. A study analyzed 115 patients with abdominal infections who were treated with laparoscopic surgery or open surgery. Its results showed that the incidence of postoperative sepsis was no different between the two groups (Balagué Ponz & Trias, 2001). Another study demonstrated that the CO_2_ pneumoperitoneum could reduce the inflammatory response in an animal model of peritonitis with respect to intraperitoneal cytokines, white blood cell count, and clinical correlates of sepsis, indicating that CO_2_ pneumoperitoneum is a protective mechanism in this condition. On the other hand, the pneumoperitoneum produced hyper-carbonic acidosis in septic animals (Araújo Filho et al., 2006). Scant data exist regarding the effect of laparoscopy and increased intra-abdominal pressure on sepsis and physiological outcome, and further studies are needed to verify its benefits or potential outcomes.

### Artificial intelligence

Advances in artificial intelligence have offered a new approach to clinical diagnosis, which has been exploited in the early predictions of sepsis (He et al., 2020; Lauritsen et al., 2020). Bourcier, Klug & Nguyen (2021) described its prospective use in the early diagnosis of NOMI, representing a machine learning model that may refine the accuracy and speed of diagnosis in critically ill patients. However, significant work is still needed to collect datasets on diagnosed NOMI patients, develop the model, and validate it in a clinical setting.

## Conclusions

NOMI is an increasingly common medical emergency and reducing its mortality remains a challenge. However, MDCT is considered to be the most valuable tool in diagnosis, despite some limitations in patients with intra-abdominal sepsis. Intra-abdominal sepsis is a high-risk factor for NOMI, and its presence may lead to more difficulty in obtaining a diagnosis and a higher mortality rate. With the growing number of abdominal operations, NOMI induced by abdominal sepsis is a complication that should not be overlooked.

## Additional Information and Declarations

### Data Availability

The following information was supplied regarding data availability: This is a literature review.
